# Novel mechanism of enhancing IRE1α-XBP1 signalling via the PERK-ATF4 pathway

**DOI:** 10.1038/srep24217

**Published:** 2016-04-07

**Authors:** Akio Tsuru, Yasutaka Imai, Michiko Saito, Kenji Kohno

**Affiliations:** 1 Laboratory of Molecular and Cell Genetics, Graduate School of Biological Sciences, Nara Institute of Science and Technology, 8916-5 Takayama, Ikoma, Nara 630-0192, Japan

## Abstract

Mammalian inositol-requiring enzyme 1α (IRE1α) is the most conserved of all endoplasmic reticulum (ER) stress sensors, which includes activating transcription factor (ATF) 6 and double-stranded RNA-dependent protein kinase (PKR)-like ER kinase (PERK). IRE1α has been known to splice *X-box binding protein 1 (XBP1)* mRNA, which is induced by ATF6 under ER stress. This spliced *XBP1* mRNA is translated into the active transcription factor that promotes the expression of specific genes to alleviate ER stress. Herein, we report that in addition to the induction of XBP1 expression by ATF6, IRE1α expression is induced by ATF4, which is downstream of PERK, under ER stress. Increased IRE1α expression results in a higher splicing ratio of *XBP1* mRNA. This effect was not transient and affected not only the intensity but also the duration of the activated state of this pathway. These multiple regulatory mechanisms may modulate the response to various levels or types of ER stress.

The endoplasmic reticulum (ER) is the site of synthesis and maturation of secretory and membrane proteins. In the ER, nascent or newly synthesized peptides fold and assemble with the aid of chaperones and folding enzymes^1^. However, not all peptides can fold properly; such failure causes the accumulation of unfolded proteins in the ER, also known as ER stress. Under ER stress, signalling pathways collectively termed as the unfolded protein response (UPR) are activated^2^,^3^,^4^,^5^.

Three principal branches of the UPR have been identified, signalling in which is initiated by the transmembrane stress sensors–activating transcription factor (ATF) 6, inositol-requiring enzyme 1 (IRE1), and double-stranded RNA-dependent protein kinase (PKR)-like ER kinase (PERK), respectively. ATF6 is originally expressed as a type II transmembrane protein, but under ER stress, its cytosolic amino terminal segment is released by proteolysis and functions as a transcription factor that induces UPR target genes including those encoding chaperones and other transcription factors such as X-box binding protein 1 (XBP1). IRE1 is conserved from yeast to mammals and possesses a luminal sensor domain^6^,^7^,^8^,^9^,^10^ and a cytosolic effector domain^11^,^12^ containing kinase and RNase subdomains. Mammals have two IRE1 paralogues, IRE1α 13 and IRE1β^14^,^15^. Of these, IRE1α is ubiquitously expressed and is required for unconventional splicing of *XBP1* mRNA^16^,^17^. The translation product of the spliced *XBP1*, XBP1s, induces UPR target genes such as those encoding chaperones and components of ER associated degradation (ERAD)^18^,^19^. PERK possesses a luminal sensor domain and a cytosolic effector domain with kinase activity. During ER stress, PERK phosphorylates eukaryotic initiation factor 2α (eIF2α), which attenuates general translation but induces the selective translation of ATF4 that up-regulates UPR target genes^20^,^21^.

The branches of the UPR described above have been studied for years, but communication among these branches has not been sufficiently investigated. In this study, we found that IRE1α was induced by ER stress both in vivo and in cultured cells. Using various knockout cells, we revealed that the increase in IRE1α expression depended on PERK-ATF4 pathway. PERK knockout decreased the ratio of *XBP1* mRNA splicing. These findings indicated that PERK-ATF4 pathway affected the efficiency of *XBP1* mRNA splicing by regulating IRE1α expression.

## Results

### IRE1α expression is induced by ER stress

**Figure 1:**
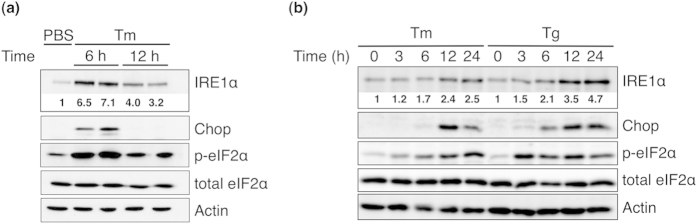
Induction of IREα expression by ER stress. (**a**) Tunicamycin (500 ng/g) was injected intraperitoneally into mice. After the treatment for the indicated periods, each excised mouse liver was lysed and analysed by western blot with specific antibodies. (**b**) HepG2 cells were treated with tunicamycin (Tm, 2 μg/ml) or thapsigargin (Tg, 0.2 μg/ml) for the indicated periods and were then lysed and analysed by western blot. Values under IRE1α bands indicate the fold-increase normalized with actin expression.

To investigate the changes in IRE1α during ER stress, the ER-stress inducer tunicamycin was injected intraperitoneally into mice. After 6 or 12 h, the mouse livers were excised and lysed. Western blot analysis (Fig. 1a) showed that Chop expression increased markedly at 6 h after injection and decreased to the initial level at 12 h. Phosphorylated eIF2α also increased at 6 h and decreased at 12 h after injection, while total eIF2α remained constant. These results indicate that tunicamycin induced ER stress in the mouse liver since induction of Chop, and phosphorylation of eIF2α are well-known ER-stress markers. Unexpectedly, IRE1α expression also increased and decreased in the same manner, suggesting that IRE1α is also an ER-stress inducible protein.

For further analysis, we examined whether ER stress could induce IRE1α expression in cultured cells. Human hepatoma HepG2 cells were treated with tunicamycin or thapsigargin and were analysed by western blot (Fig. 1b). Phosphorylation of eIF2α increased slightly after 3 h of treatment with tunicamycin and reached the highest level at 24 h. Chop expression started to increase at 6 h and reached a maximum at 12 h before slightly decreasing. IRE1α expression also increased by 12 h of treatment and remained high up to 24 h. The effects of thapsigargin were observed sooner and lasted longer than those of tunicamycin. Phosphorylated eIF2α increased markedly after 3 h of incubation and remained at an almost constant level until the end of the experiment (24 h). Chop expression started to increase after 3 h of treatment and reached a maximum at 12 h. IRE1α expression was induced at 6 h after treatment and reached a maximum between 12 and 24 h of treatment. Together, these observations indicate that ER stressors could induce IRE1α expression in HepG2 cells.

### IRE1α expression is transcriptionally regulated

**Figure 2:**
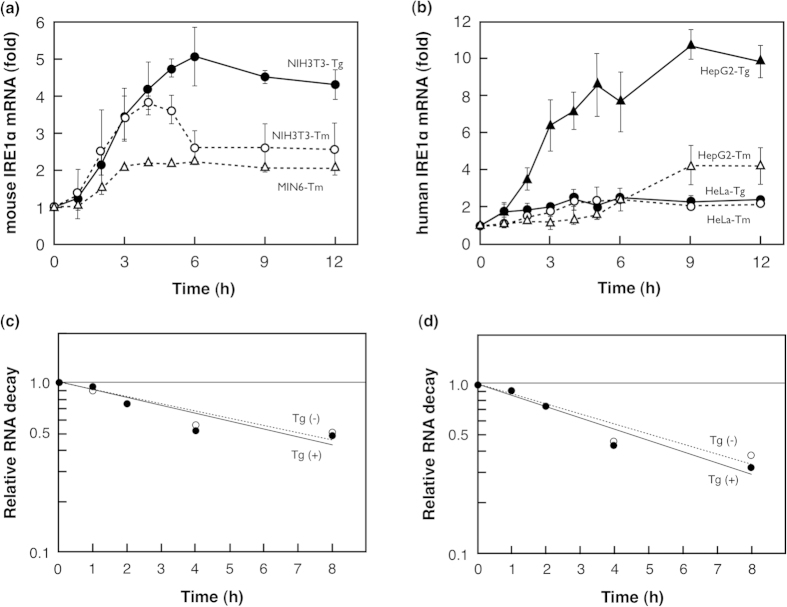
Transcriptional regulation of IRE1α mRNA expression. Cells were treated with tunicamycin (Tm) or thapsigargin (Tg) for the indicated periods. IRE1α mRNA from mouse (**a**) or human (**b**) cells was quantified by qRT-PCR. Data presented are the average of three independent experiments, with standard deviation indicated by error bars. Stability of IRE1α mRNA in mouse NIH3T3 (**c**) and human HeLa cells (**d**). Cells treated with (+) or without (−) thapsigargin, were incubated in media containing actinomycin D for the indicated periods. RNA was extracted and analysed by qRT-PCR. Relative RNA decay is expressed as the *IRE1α* mRNA/*GAPDH* mRNA ratio, and values at time 0 were set to 1. Error bars indicate standard deviation.

Because the expression of ER-stress inducible proteins, e.g. chaperones, is usually regulated at the transcriptional level, we examined whether *IRE1α* mRNA expression increases in mouse (Fig. 2a), and human cells (Fig. 2b) under ER stress by using qRT-PCR.

*IRE1α* mRNA expression was most increased in HepG2 cells by both tunicamycin and thapsigargin treatments compared to that in all cells tested: *IRE1α* mRNA expression increased gradually and reached a maximum (4-fold increase) after 9 h of tunicamycin treatment, and started to increase after 2 h of thapsigargin treatment and reached a maximum (about 10 fold) at 9 h before slightly decreasing. The increases in *IRE1α* mRNA expression were also observed in mouse fibroblast NIH3T3, mouse insulinoma MIN6, and human adenocarcinoma HeLa cells, which were over 2-fold.

To exclude the possibility that *IRE1α* mRNA expression is regulated by degradation, the stability of *IRE1α* mRNA was studied. NIH3T3 cells and HeLa cells were incubated in media with or without thapsigargin for 3 h, and actinomycin D was added. After 0–8 h of incubation with actinomycin D, cellular RNA was extracted and analysed by qRT-PCR (Fig. 2c,d). Half-lives of *IRE1α* mRNA from NIH3T3 cells treated with or without thapsigargin were calculated as 6.6 h and 7.2 h, respectively. Half-lives of *IRE1α* mRNA from HeLa cells treated with or without thapsigargin were calculated as 4.5 h and 5.1 h, respectively. Because differences in half-lives of *IRE1α* mRNAs between treated and non-treated cells were considered insufficient to explain the increase in mRNA expression due to ER stress, the increase of IRE1α mRNA expression may be due to transcriptional up-regulation in both mouse and human cells.

### ATF4 up-regulates IRE1α expression

**Figure 3:**
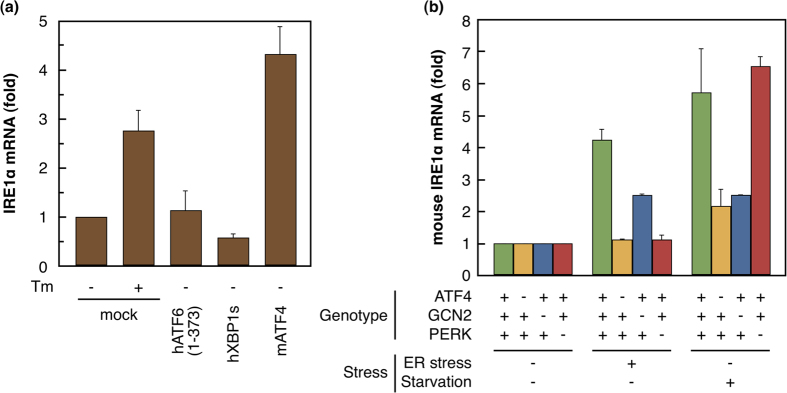
ATF4-dependent induction of IRE1α. (**a**) Transcription factors were over-expressed transiently in HeLa cells; *IRE1α* mRNA expression was quantified by qRT-PCR and normalized with *GAPDH* expression. The value for mock without tunicamycin (Tm) treatment was set as 1. Data presented are the average of three independent experiments, with standard deviation indicated by error bars. hATF6 (1–373), cytosolic portion of human ATF6α; hXBP1s, spliced human XBP1; mATF4, mouse ATF4. (**b**) Mouse fibroblasts with various genotypes were incubated under stress or non-stress conditions. ER stress was induced by incubating cells in medium containing 2 μg/ml Tm for 9 h. Amino acid starvation was induced by incubating cells in Hanks’ solution for 6 h. *IRE1α* mRNA expression was quantified by qRT-PCR and normalized with *GAPDH* expression; values for non-stressed cells were set as 1. The basal expressions in PERK^−/−^, ATF4^−/−^, and GCN2^−/−^ cells were 0.70, 1.09, 1.00-fold of that in wild type cells, respectively. Data presented are the average of three independent experiments, with standard deviation indicated by error bars.

To identify transcription factors that up-regulate IRE1α expression, XBP1s, ATF4, or the cytosolic portion of ATF6 was over-expressed in HeLa cells. One day after transfection, cellular RNA was extracted. qRT-PCR analysis showed that over-expression of ATF4 in HeLa cells markedly increased IRE1α mRNA expression, whereas over-expression of the other factors had no effect (Fig. 3a). These observations indicated that ATF4 could induce the expression of *IRE1α* mRNA.

It is well known that ATF4 is induced in a PERK-dependent manner under ER stress and in a general control nonderepressible 2 (GCN2)-dependent manner under amino acid starvation^20^. Therefore, we studied the induction of *IRE1α* mRNA by qRT-PCR under stresses in mouse embryonic fibroblasts (MEF) lacking ATF4, PERK, or GCN2 (Fig. 3b). When treated with tunicamycin, ATF4-knockout MEFs and PERK-knockout MEFs showed no increase in *IRE1α* mRNA expression. In contrast, IRE1α mRNA expression in wild type (WT) and GCN2-knockout MEFs considerably increased. However, under amino acid starvation, IRE1α mRNA expression was little induced in both GCN2-knockout and ATF4-knockout MEFs compared with that in WT and PERK-knockout MEFs, which showed a marked increase. These observations suggest that the induction of IRE1α under stress is mainly regulated by ATF4.

### Up-regulation of IRE1α affects the efficiency of *XBP1* mRNA splicing

**Figure 4:**
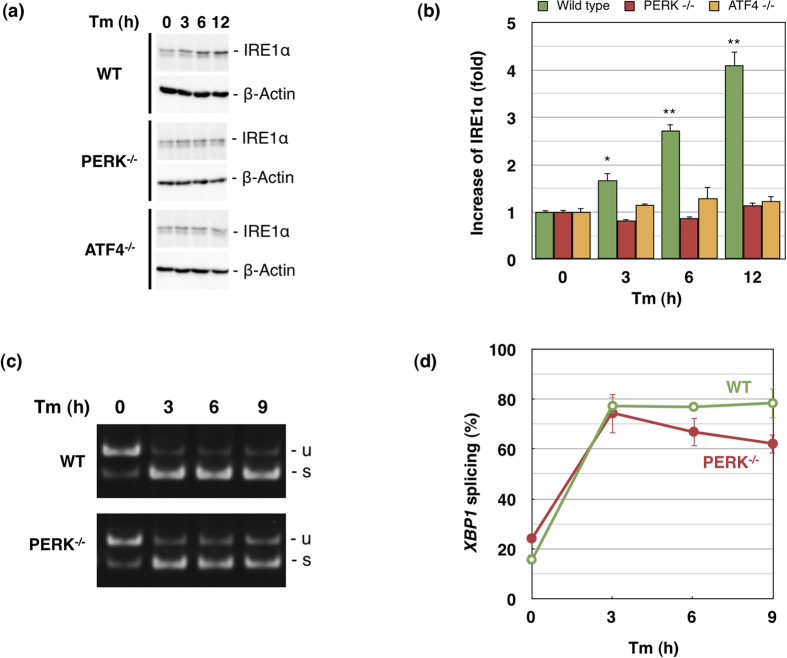
Increase in IRE1α expression affects the efficiency of *XBP1* splicing. Mouse fibroblasts of various genotypes were incubated with tunicamycin (2 μg/ml) for the indicated periods. (**a**) IRE1α expression was detected by western blot. (**b**) Western blot was repeated 3 times and IRE1α bands were quantified and graphed; values for non-stressed cells were set as 1. IRE1α was significantly increased in wild type cells (*p < 0.05, **p < 0.0001), and significant differences were observed between wild type and other cells, PERK^−/−^ and ATF4^−/−^ after tunicamycin treatment (p < 0.05). The basal expressions in PERK^−/−^and ATF4^−/−^ cells were 0.89 and 1.02-fold of that in wild type cells, respectively. (**c**) Splicing of *XBP1* mRNA was detected by electrophoresis after RT-PCR. (**d**) Splicing ratio of *XBP1* mRNA was quantified by qRT-PCR. Significant differences were observed between wild type and PERK^−/−^ at 6 h and 9 h (n = 3, p < 0.05). Error bars indicate standard deviation. u, unspliced *XBP1*; s, spliced *XBP1*. [XBP1 splicing (%)] = [XBP1s]/([total XBP1]) ×100.

To explore the regulation of IRE1α expression by ATF4, we examined whether an increase in IRE1α expression affected ER stress signalling. First, we confirmed whether the increase of *IRE1α* mRNA expression caused by ER stress was reflected by its protein level in MEFs. WT, ATF4^−/−^, and PERK^−/−^ MEFs were treated by tunicamycin and then lysed and analysed by western blot (Fig. 4a), and IRE1α bands were quantified and graphed (Fig. 4b). As expected, the IRE1α protein content in WT MEF increased over time and reached over 4-fold its initial value after 12 h of the treatment. In contrast, PERK^−/−^ and ATF4^−/−^ MEFs showed little or no increase in IRE1α protein content.

The efficiency for *XBP1* splicing in these cells was also examined by RT-PCR. After tunicamycin treatment, cells were collected and cellular RNA was extracted. PCR products were electrophoresed (Fig. 4c). We also performed qRT-PCR to quantify the splicing ratio of *XBP1* mRNA (Fig. 4d). In WT cells, *XBP1* splicing ratio increased from approximately 20% to 80% after 3 h of treatment with tunicamycin, and this level was maintained until the end of the experiment (9 h). In PERK^−/−^ cells, the splicing ratio increased to over 75%, which is a little less than that in WT cells, after 3 h of treatment with tunicamycin; however, in contrast to that in WT cells, the splicing ratio gradually decreased thereafter. These results suggest that the PERK-ATF4 pathway plays an important role in the increase of signalling by the IRE1α-XBP1 pathway, especially by sustaining the increased *XBP1* mRNA splicing ratio.

## Discussion

IRE1 is the most conserved ER stress sensor as it is found organisms from yeast to mammals. While Ire1p (IRE1 in yeast) is the only ER stress sensor in yeast cells, mammals have additional ATF6 and PERK that sense ER stress. It is well known that activated ATF6 induces the transcription of *XBP1*^16^,^22^ and of other UPR target genes. *XBP1* mRNA is spliced by activated IRE1α and a ligase complex^23^ that includes RtcB^24^,^25^,^26^. Because IRE1α can splice a very small amount of *XBP1* mRNA without the induction by ATF6^16^, ATF6 has largely been considered to affect signalling by the IRE1α-XBP1 pathway. In this study, we found that IRE1α expression is induced by the PERK-ATF4 pathway, suggesting that the activity of IRE1α is maintained at low levels in steady-state cells.

It has been reported that yeast cells that do not have ATF6, PERK, or ATF4 genes, have alternative mechanisms to maintain low activity of Ire1p under non-stress conditions^27^. Yeast Ire1p contains a non-conserved subdomain, Subregion I, at the N-terminus^6^ that does not exist in its mammalian counterpart. This N-terminal subdomain was reported to be intrinsically disordered and to act as an intramolecular suppressor under non-stress conditions^27^. Taken together, the low activity of Ire1p and IRE1α pathways during steady-state must be advantageous for both yeast and mammalian cells.

It has been reported that IRE1α cleaves a wide variety of mRNAs besides *XBP1* mRNA by the regulated IRE1-dependent decay (RIDD) mechanism that involves the relatively promiscuous degradation of membrane-associated mRNAs under severe stress conditions^28^,^29^,^30^, and moreover, RIDD induces apoptosis^30^. With regard to this, Lee *et al*. have reported that IRE1α was increased and activated in the *XBP1*-deleted liver^31^. Furthermore, Hur *et al*. reported that such increased and activated IRE1α induces RIDD activity^32^. These observations suggest that the ratio of IRE1α protein to *XBP1* mRNA is critical in biological processes; consequently, to suppress apoptosis-inducible RIDD activity in steady-state cells, IRE1α is down-regulated when *XBP1* mRNA expression is low.

**Figure 5:**
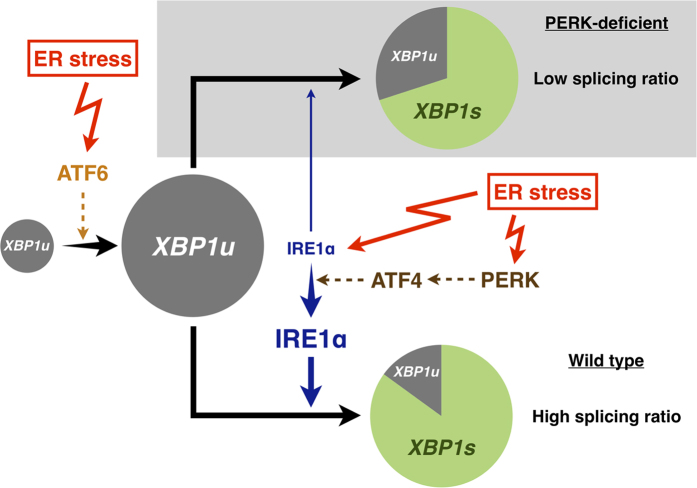
ATF6 and PERK increases *XBP1s* mRNA expression in different ways. Under ER stress, ATF6 promotes the transcription of *XBP1* gene, and the transcript *XBP1u* mRNA is spliced by IRE1α to produce *XBP1s* mRNA. PERK induces ATF4 translation, which induces IRE1α expression (in this paper). Increased IRE1α protein improves the efficiency of *XBP1* mRNA splicing (in this paper). In other words, PERK increases the ratio of *XBP1s* to *XBP1u*, whereas ATF6 increases the sum of *XBP1u* and *XBP1s.*

The splicing ratio of *XBP1* mRNA in PERK^−/−^ cells was increased by tunicamycin treatment but decreased thereafter, whereas PERK-expressing cells maintained this increased ratio for several hours. Therefore, it appears that PERK contributes to the IRE1α-XBP1 pathway in a manner different to that of ATF6. These differences might be caused by PERK and ATF6 utilizing two different mechanisms to increase *XBP1s* mRNA expression (Fig. 5): ATF6 increases *XBP1s* mRNA expression by increasing *XBP1* transcription, while PERK increases *XBP1s* mRNA expression by increasing IRE1α expression, which consequently increases splicing efficiency of *XBP1* mRNA.

In this study, we reported a novel mechanism of enhancing IRE1α-XBP1 signalling via the PERK-ATF4 pathway in addition to ATF6. Together with previously studied mechanisms^16^, it can be concluded that expression of IRE1α and its substrate *XBP1* mRNA was regulated separately by ATF4 and ATF6. This may enable cells or organisms to cope with various types and intensities of stress by modulating the output of the IRE1α-XBP1 pathway.

However, it could not be elucidated whether ATF4 promoted IRE1α expression directly or indirectly in this study. Downstream of ATF4 contains several transcription factors such as ATF3 and CHOP. The possibilities that homodimers or heterodimers of those factors with other b-zip proteins^21^ promote IRE1α expression has not been excluded. Solving detailed mechanism for inducing IRE1α expression will help us understand the refined strategy of cells against ER stress.

## Methods

### Animals

C57BL/6J mice and a New Zealand white rabbit were purchased from CLEA Japan (Tokyo, Japan). All experimental protocols involving animals were approved by the Committee on Animal Research at Nara Institute of Science and Technology (NAIST), and were carried out in accordance with the institutional guidelines of NAIST.

### Antibodies

Anti-IRE1α was prepared as follows. cDNA encoding the cytosolic domain (1513–3054) corresponding to amino acids 465–977 of mouse IRE1α (GenBank Accession No. NM023913) was amplified using pcDNA3.1 (+) mIRE1α 33 as a template and 5′-ACATGCATGCACTTACCCCCTGAGCGTG-3′ and 5′-ACGCGTCGACTCAGAGGGCATATGGAATCACT-3′ as primers. The amplified DNA was digested with SphI and SalI and cloned into a pQE80L vector (Qiagen, Hilden, Germany) to produce the His-tagged protein in *Escherichia coli*. Antigens were purified as previously described^34^. For immunization, a rabbit was injected with an emulsion containing 0.2 mg antigen and Sigma Adjuvant System (Sigma-Aldrich, St. Louis, MO), for 4 times with 3-week intervals between injections; antiserum was collected at 10 days after the final injection. Anti-C/EBP homologous protein (CHOP), anti-eukaryotic initiation factor 2α (eIF2α), anti-phospho-eIF2α, and anti-β-actin were purchased from Affinity BioReagents/Thermo Fisher Scientific (Waltham, MA), Cell Signaling Technology (Danvers, MA), Biosource (Camarillo, CA), and Novus Biologicals (Littleton, CO), respectively. Horseradish peroxidase-conjugated anti-mouse IgG and anti-rabbit IgG were purchased from DAKO (Glostrup, Denmark) and GE Healthcare (Little Chalfont, UK), respectively.

### Cell culture, treatment, and transfection

All cells, except mouse embryonic fibroblasts (MEFs), were cultured in DMEM supplemented with 10% FCS in 5% CO_2_/air at 37 °C. MEFs were established in Dr. D. Ron’s laboratory (University of Cambridge, Cambridge, UK)^20^ and were donated by Dr. S. Takahashi (Wild type, PERK^−/−^, and GCN2^−/−^) (Tokyo University of Pharmacy and Life Sciences, Tokyo, Japan)^35^ and Dr. T. Ishihara (ATF4^−/−^) (Nihon University, Tokyo, Japan)^36^. MEFs were cultured in DMEM supplemented with 10% FCS, 55 μM 2-mercaptoethanol, and nonessential amino acids^20^. To induce ER stress, HeLa cells were treated with 0.5 μg/ml tunicamycin or 0.05 μg/ml thapsigargin. Other cells were treated with 2 μg/ml tunicamycin or 0.2 μg/ml thapsigargin. Cells were washed 3 times with PBS then lysed in Laemmli sample buffer for SDS-PAGE^37^ by sonication. RNA was extracted from cells by using RNAiso plus (Takara Bio, Otsu, Japan) according to the manufacturer’s protocol. Cloning of cDNA encoding cytosolic portion of human ATF6α into mammalian expression vector pCAX was described previously^38^. Human XBP1s and mouse ATF4 cDNAs were also inserted into pCAX by the same cloning method. Plasmids were transfected using Lipofectamine 2000 (Invitrogen/Thermo Fisher Scientific, Waltham, MA) according to the manufacturer’s protocol.

### Drug treatment in mice

Tunicamycin (500 ng/g body weight) was injected into 10-week-old mice intraperitoneally^39^. At 6–12 hours after injection, mice were sacrificed and their livers were excised. After washing three times in Hanks’ solution, tissues were homogenized using a Polytron homogenizer (Kinematica AG, Luzern, Switzerland) in Laemmli sample buffer. Insoluble materials were centrifuged, and the supernatant was collected.

### Western blot

After SDS–PAGE, proteins were electrotransferred onto a PVDF membrane (MERCK Millipore, Billerica, MA). Proteins on the membrane were immunodetected using specific antibodies.

### RNA stability assay

After 3 h of thapsigargin treatment, actinomycin D (2 μg/ml) was added to cell cultures. At various time points, RNA was extracted using RNAiso plus. Obtained RNA was quantitatively analysed as described below.

### RT-PCR

After synthesis of cDNA from total RNA using SuperScript II (Invitrogen/Thermo Fisher Scientific), we performed quantitative real-time PCR (qPCR) in triplicate by using a Light-Cycler 480 (Roche, Basel, Switzerland) with the following primers:

human GAPDH (Accession No. BC023632), 5′-AGCCACATCGCTCAGACAC-3′ and 5′-CGCCCAATACGACCAAAT-3′; mouse IRE1α (Accession No. NM_023913), 5′-GCCGAAGTTCAGATGGAATC-3′ and 5′-ATCAGCAAAGGCCGATGA-3′; human IRE1α (Accession No. NM_001433), 5′-GCCGAAGTTCAGATGGAATC-3′ and 5′-ATCTGCAAAGGCCGATGA-3′; mouse XBP1 (Accession No. NM_013842), 5′-GAATGGACACGCTGGATCCT-3′ and 5′-GCCACCAGCCTTACTCCACTC -3′; and spliced form of mouse XBP1, 5′-GAGTCCGCAGCAGGTG-3′ and 5′-GTGTCAGAGTCCATGGGA-3′.

The splicing of mouse *XBP1* mRNA was also analysed by electrophoresis after PCR with the following primers:

5′-GAGAACCAGGAGTTAAGAACACG-3′ and 5′-GAAGATGTTCTGGGGAGGTGAC-3′.

### Quantification and statistical analysis

Bands on gels and membranes were quantified using LAS-4000 and Multigauge software (Fujifilm, Tokyo, Japan). Quantified values obtained from qRT-PCR and LAS-4000 were statistically analysed using Microsoft Excel software (Microsoft, Redmond, WA).

## Additional Information

**How to cite this article**: Tsuru, A. *et al*. Novel mechanism of enhancing IRE1α-XBP1 signalling via the PERK-ATF4 pathway. *Sci. Rep.* **6**, 24217; doi: 10.1038/srep24217 (2016).
